# The effect of the health poverty alleviation project on financial risk protection for rural residents: evidence from Chishui City, China

**DOI:** 10.1186/s12939-019-0982-6

**Published:** 2019-05-31

**Authors:** Chu Chen, Jay Pan

**Affiliations:** 1 West China School of Public Health and West China Fourth Hospital, Sichuan University, No. 16, Section 3, Ren Min Nan Road, Chengdu, 610041 Sichuan China; 2 West China Research Center for Rural Health Development, Sichuan University, No. 17, Section 3, Ren Min Nan Road, Chengdu, 610041 Sichuan China

**Keywords:** Poverty, Health policy, Policy analysis, Financial risk sharing, Illness burden, China

## Abstract

**Background:**

Illness is the leading cause (44%) of poverty in China. Since 2016, The health poverty alleviation project, an integral component of the Targeted Poverty Alleviation in China, was implemented in 2016 to strengthen financial risk protection against illness for financially backward segments of the population. However, the effects of the health poverty alleviation project on financial risk protection have not been explored in the literature, this paper aims to bridge the gap.

**Methods:**

Using panel data on 63,426 rural households in Chishui City, China, from 2014 to 2017, the difference-in-differences with propensity score matching method was employed.

**Results:**

The health poverty alleviation project reduces out-of-pocket payments by 15% on average and decreases the probability of catastrophic health expenditure (annual out-of-pocket payments exceeding 10% of annual household income) and impoverishing health spending occurrence (out-of-pocket payments are forcing a household into poverty or into deeper poverty) by 7.7 and 11.7%, respectively. Additionally, the project increases the number of annual hospitalizations per household by 0.035.

**Conclusion:**

Our study demonstrates that the health poverty alleviation project significantly improves financial risk protection by reducing out-of-pocket payments and decreasing the probability of incurring catastrophic or impoverishing levels of health expenditure. Our study has implications for the poverty reduction policies and reform of the Chinese health financing system.

## Introduction

The burden imposed by out-of-pocket (OOP) payments for medical treatments results in financial hardship for millions of people in low-and middle-income countries when they seek health care [1–3]. The financial burden of health care often forces people to choose between satisfying their basic needs—such as food and education—and purchasing health care plans to save loved ones from illness, suffering, and shortened life spans [4]. It is estimated that almost 100 million people are pushed into extreme poverty each year because of high OOP payments [5].

Various interventions attempting to eliminate poverty arising from high OOP payments and to provide financial risk protection to the poor have been introduced by governments and nongovernmental organizations (NGOs). Some countries, Indonesia and Thailand for example, offer public health insurance to the poor without requiring any co-payment [6, 7]. Other countries for instance, Vietnam, do provide a public health insurance, but the poor are still required to make a co-payment of 5% of the health care costs [8], while China, provides voluntary health insurance, for which the government subsidizes a substantial part of the premium (more than 80%) [9]. The patients are required to co-pay of 25–50% of the health care costs [6, 9–11]. Another method of health funding offered by other countries, for example, Georgia, is to arrange cash transfers to poor households to meet their health care needs [12]. NGOs such as Health Poverty Action offer consulting services to strengthen health services in marginalized communities in developing countries. The common justification for intervention is that illness and poverty create a vicious cycle in which ill health maintains poverty, while poverty leads to ill health [13]. However, the interventions do not always achieve their goal of significantly reducing the financial burden for the poor [14–17].

China has made dramatic progress in reducing domestic poverty. Over the past three decades, 700 million people have been lifted out of poverty in China [18]. However, by the end of 2015, China still had 55.75 million people living below the national poverty line (defined as living on less than approximately $1 a day). Aiming to end extreme poverty by 2020, the Central Government of the People’s Republic of China launched the “Targeted Poverty Alleviation” project in 2015. Since then, a series of poverty-relief programs have been launched [19].

Illness is an important cause of poverty in China, and studies estimate that for between 7.5 and 44% of people in poverty, illness is the root cause [20–22]. Considering that, the Central Government developed the health poverty alleviation project as an integral component of its Targeted Poverty Alleviation, the project has the clear aim of strengthening financial risk protection against health shocks and illness for poor people in rural China [23]. Since the implementation of the health poverty alleviation project in 2016, more than 4.2 million poor patients with serious or chronic diseases have been treated [24]. However, to the best of our knowledge, the effect of the health poverty alleviation project on financial risk protection has never been explored in the literature.

This paper aims to fill this gap by assessing the impact of the project on financial risk protection in China. Using administrative data from Chishui, this study contributes to the literature by providing empirical evidence on the effect of the health poverty alleviation project on financial risk protection in rural China. In doing so, we extend the international literature on poverty alleviation by strengthening financial risk protection against illness.

## Background

**Fig. 1:**
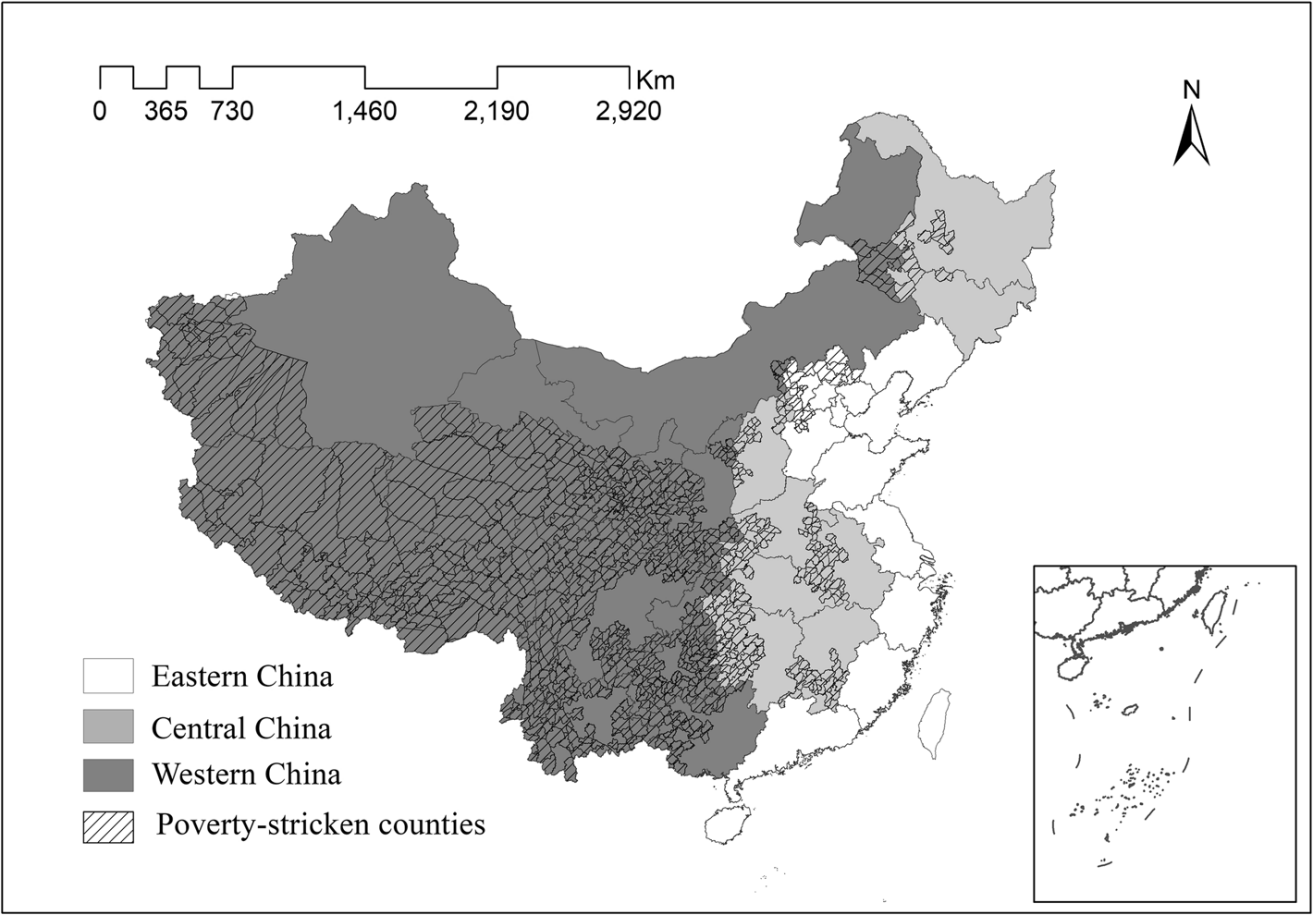
The distribution of poverty-stricken counties in 14 extreme poverty regions. Source:The State Council Leading Group Office of Poverty Alleviation and Development. The publication of the poverty-stricken counties in 14 extreme poverty regions. 2012

Over the past three decades, the Chinese government has implemented a series of actions to combat poverty in rural China, for example, by providing subsidized loans to the poor, removing agricultural taxes, and building roads [25]. In 2012, the government defined 14 extreme poverty regions, including 680 poverty-stricken counties, and established supportive polices for them (see Fig. 1) [26]. By the end of 2015, there were still 55.75 million people living below the national poverty line, and the incidence of poverty in Eastern, Central, and Western China was 1.8, 6.2, and 10%, respectively [19].

In November 2015, the CPC Central Committee and the State Council issued the “Decision on Winning the Fight Against Poverty,” which charting the course of the Chinese poverty alleviation campaign till 2020. Following this, 118 related policy documents and implementation plans were released by various national government departments. In December 2016, the 13th Five-Year (2016–2020) National Plan for Poverty Reduction was formulated, setting explicit objectives and specific tasks for poverty reduction over those 5 years [27].

The Chinese government is progressing on the poverty alleviation project with unprecedented vigor. Unlike previous approaches, the new national poverty reduction project in China precisely identifies poor households and draws up personalized plans for each household’s poverty reduction. In addition, the project has a clear goal: to lift impoverished rural people currently living below the national poverty line out of poverty within 5 years, and lowering the poverty headcount rate of poverty-stricken counties to less than 2% (or 3% for minority counties) [28].

To guarantee progress, the Central Government developed a series of management systems. First, the chief leader of the local government shoulders the overall responsibility for the progress of poverty-reduction work. Poverty reduction is a top priority of local government in poverty-stricken regions. Second, similar to the State Council Leading Group Office of Poverty Alleviation and Development, provincial, prefectural (city), and county governments each set up a corresponding office to strengthen interdepartmental coordination regarding project implementation. The local chief leader holds the position of director of the office. Third, the State Council Leading Group Office of Poverty Alleviation and Development routinely organizes peer reviews among regions and employs a third party for a critical assessment of the poverty reduction project.

Considering illness is the leading cause of poverty in China, the health poverty alleviation project was launched in 2016. The objective of this project is to strengthen financial risk protection against illness for poor populations. This approach follows three main strategies. The first is a preventive program, such as offering free physical examinations to poor people and including them in the electronic health record (EHR) system for health management. Second, the capacity of local health institutions to provide high-quality treatment and care is enhanced by investing in county hospitals and township health centers. Generally, medical expenses at township health centers, county hospitals, and city hospitals rise in line with the price-regulation policy in China. Capacity building of local health institutions aims to ensure timely and effective treatment within the county, at relatively lower cost and without patients having to incur out-of-county traveling and accommodation expenses. Third, the project seeks to strengthen the social security system. The New Rural Cooperative Medical Scheme (NCMS) is a government-based voluntary health insurance plan for the rural population. By the end of 2015, 98.80% of China’s rural population had been enrolled in the NCMS [29]. Government subsidies are the major funding source of NCMS, while the individual only pays for a small proportion of insurance costs (for example, in 2016, in Chishui, the NCMS premium was 510 RMB per person for all rural residents, of which the government subsidized 420 RMB and the individual paid 90 RMB). The NCMS covers inpatient service as well as a small number of outpatient service, whereby the patients are required to co-pay 25–50% of the health care costs [6, 11].

Overall, the health poverty alleviation project strengthens the social security system in several ways: increasing subsidies for premiums in NCMS; extending NCMS coverage to all poor households without requiring premiums; increasing NCMS reimbursement (compensation proportion); and expanding the NCMS benefit package to include the treatment of critical illnesses and provision of special medical aid for poor families. After the implementation of the health poverty alleviation project, members of poor households could participate in NCMS at no charge and, when suffering from illness, would receive more reimbursement for the treatment than did standard NCMS members. If a family’s OOP payments are catastrophic, according to criteria defined by the local government, they would be further reimbursed by NCMS. If, despite the reimbursements from NCMS, the OOP payments still constitute a considerable portion of the family’s limited disposable income, medical aid would be provided to cover a part or all of OOP payments. The degree to which reimbursement rates and medical aid standards increase is decided by the local government, mainly based on their fiscal capacity.

## Methods

### Study area

**Fig. 2:**
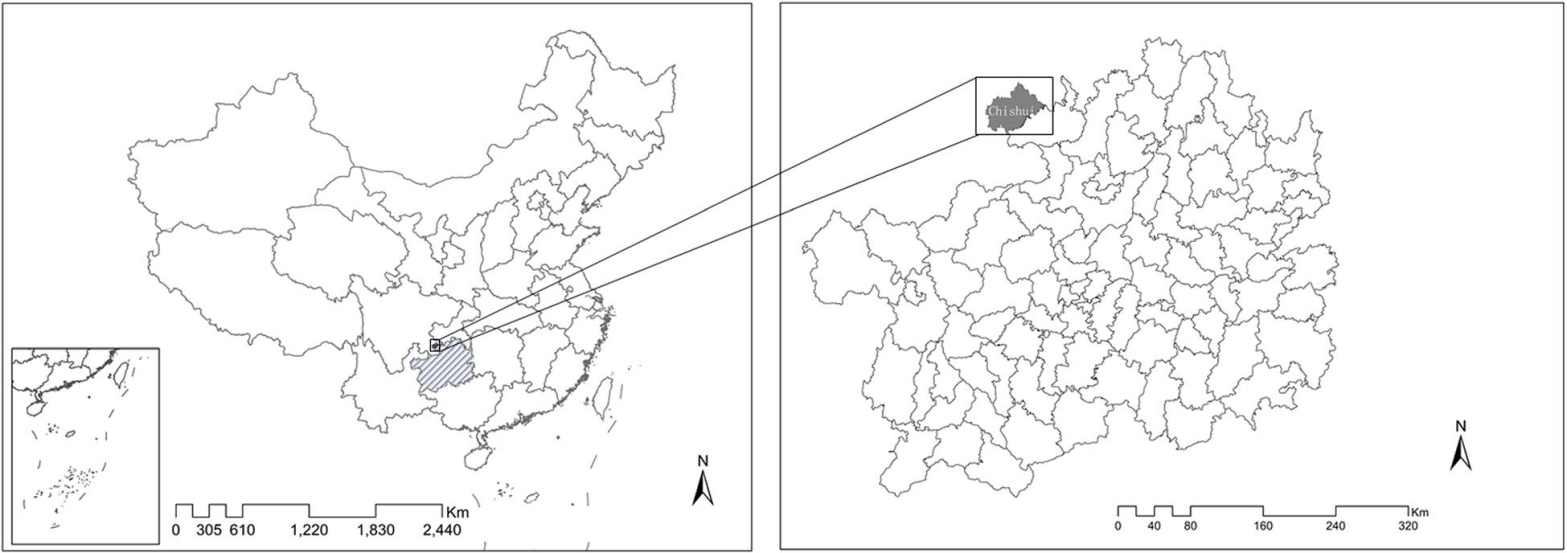
The geographic location of Chishui. Notes: The shaded area on the left-hand map shows the location of Guizhou province in China, and the shaded area on the right shows the location of Chishui in Guizhou province

Our study area is Chishui, a county-level city located in Southwest China in the northernmost county of Guizhou. Figure 2 shows its geographic location.

Chishui is a mountainous area of 1801.2 km^2^. The population of 314,118 people included 201,388 rural residents in 2015 [30]. The poor who living under the national poverty line comprise 7.5% of Chishui’s population. Among the population in poverty, 28.52% reported illness as their leading cause of poverty in Poverty Register System. In 2015, the income per capita of urban residents and rural residents was 23,938 RMB, and 9235 RMB, respectively. The unemployment rate in Chishui was 2.17%.

**Fig. 3:**
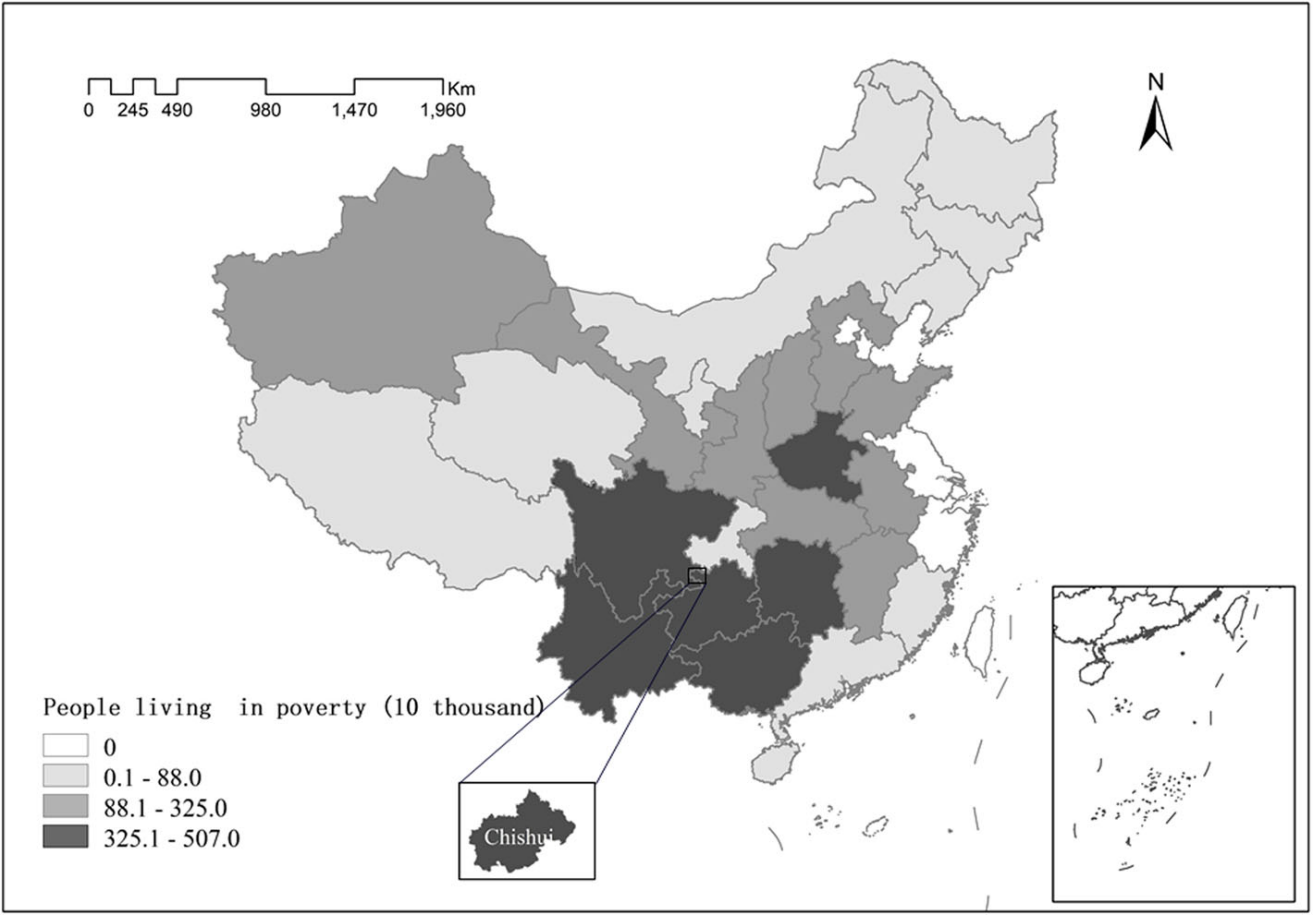
People living in poverty in each province in 2015. Source:Poverty Monitoring Report of Rural China 2016

**Fig. 4:**
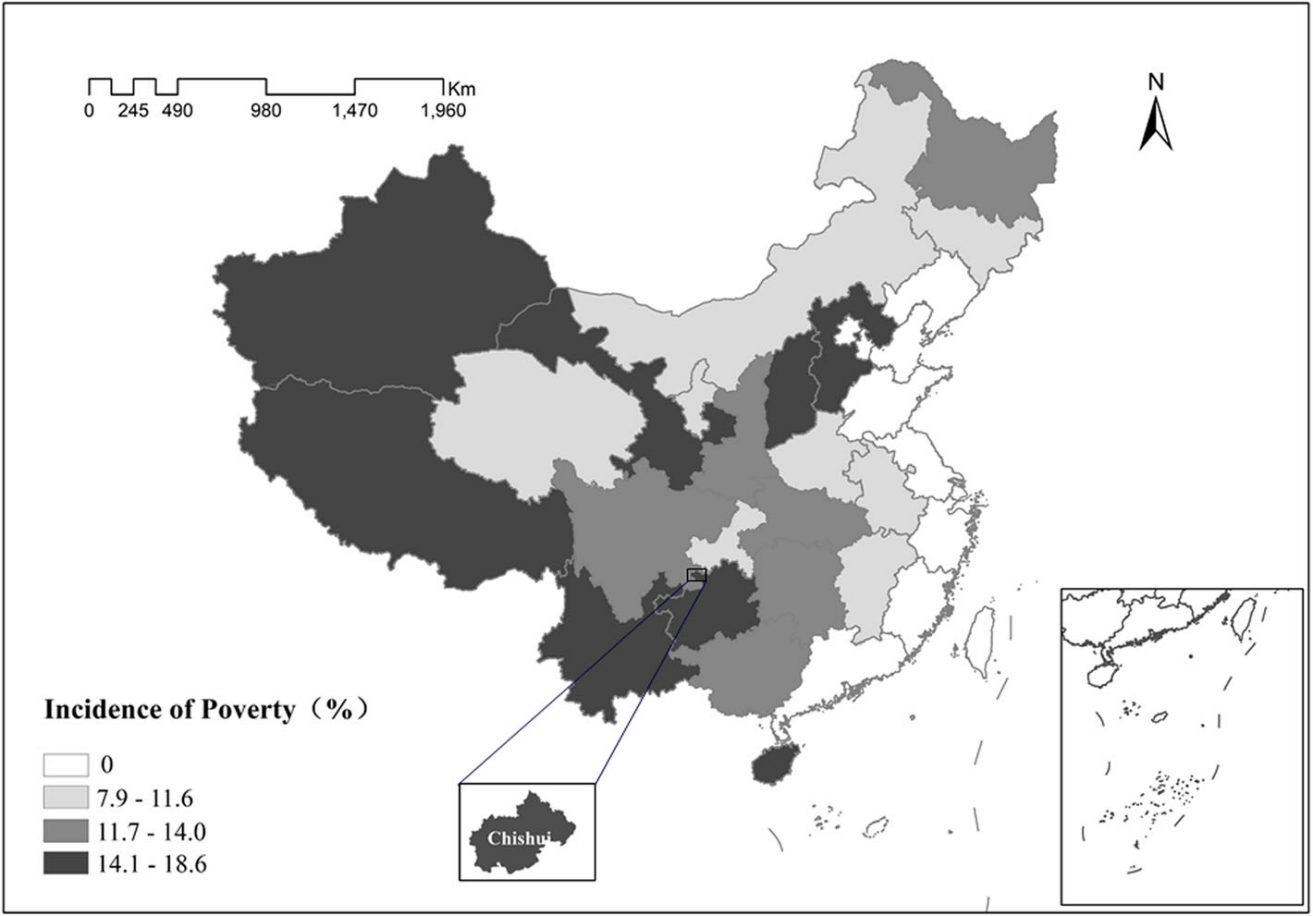
Incidence of poverty in each province in 2015. Source:Poverty Monitoring Report of Rural China 2016

We chose Chishui as our study area for the following two reasons: First, Chishui is among the 680 poverty-stricken counties identified by the Chinese government. Second, it is located in Southwest China, where prevalence of poverty is relatively high (see Fig. 3). In the absence of data on poverty incidence in all the poverty-stricken counties, we compared the incidence of poverty in Chishui with the national incidence of poverty. The incidence of poverty in Chishui in 2015 was 7.5%, higher than the national incidence of poverty (5.7%). Furthermore, we compared the incidence of poverty in each province. It demonstrates that poverty in Guizhou province, where Chishui is located, is higher than most of the provinces in China (see Fig. 4). Therefore, Chishui is representative of a typical poverty-stricken county in China to some extent.

### Study period

The health poverty alleviation project was implemented in Chishui in 2016. To evaluate the project’s impact, we chose 2014 through 2017 as our study period. 2014–2015 represents the pre-exposure period, while 2016–2017 is the postexposure period.

### Intervention

**Fig. 5:**
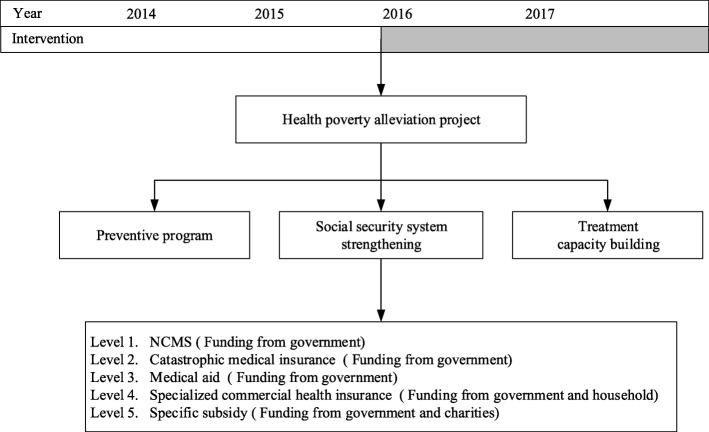
Implementation of the health poverty alleviation project in Chishui. Notes: Chishui launched the health poverty alleviation project at the beginning of 2016. The gray part of the timeline indicates the policy implementation period

Chishui launched the health poverty alleviation project to support rural households in poverty. Guided by the aforementioned three main strategies of the Central Government’s health poverty-reduction policy, the health poverty alleviation project in Chishui consists of three main parts: illness prevention, treatment capacity building, and social-security system strengthening. Figure 5 summarizes the specific interventions included in the health poverty alleviation project in Chishui.

Based on the Central Government’s policy, Chishui further extended the social security system into 5 levels as follows:

- Level 1: NCMS. In 2016, the NCMS premium for all rural residents was set at 510 RMB per person in Chishui, of which the government subsidized 420 RMB and the individual paid 90 RMB. After the implementation of the health poverty alleviation project, the premium for the poor is entirely subsidized by the government, which means any individual identified as poor does not have to pay premium to the NCMS. Furthermore, the poor will receive an additional 5% reimbursement of total inpatient costs over the regular rate. For example, if the regular reimbursement rate for inpatient costs at a township hospital is 90%, then impoverished households will receive a reimbursement of 95%.
- Level 2: catastrophic medical insurance. Households in poverty are also covered by catastrophic medical insurance at no charge. They are entitled to additional reimbursement if their co-payment is more than 3000 RMB despite the NCMS reimbursement. The reimbursement rate in the catastrophic medical insurance category is generally 50%. Thus, for example, if OOP payments are 6000 RMB after NCMS reimbursement, poor households would receive a reimbursement of 3000 RMB under the catastrophic medical insurance.
- Level 3: medical aid. If patients from a poor household cannot afford OOP payments even after receiving reimbursement from both the NCMS and catastrophic medical insurance, then medical aid will be provided up to 10,000 RMB.
- Level 4: specialized commercial health insurance. The premium for this insurance program is 60 RMB per person per year, which is subsidized by the government by 40 RMB. The beneficiary could receive compensation ranging from 1000 RMB to 30,000 RMB depending on the individual case. This reimbursement targets inpatients facing sudden illness or accidents.
- Level 5: special subsidy. If an impoverished household still cannot afford copayments after the first four levels of reimbursements, a special subsidy would be offered by the Grant of Medical Assistance for Poverty. These grants are jointly funded by the Chishui government and various charities and has been offered since the initiation of the health poverty alleviation project. The subsidy amount is determined as follows:

1 $$ \mathrm{Special}\ \mathrm{subsidy}=\left\{\begin{array}{c} Threshold\times S\mathrm{ize}\hbox{-} \left(\mathrm{Income}\hbox{-} \mathrm{OOP}\right),\mathrm{if}\ \mathrm{Income}\hbox{-} \mathrm{OOP}\ge 0\\ {} Threshold\times S\mathrm{ize},\mathrm{if}\ \mathrm{Income}\hbox{-} \mathrm{OOP}<0\end{array}\right. $$

*Threshold* is the income threshold for the poverty line as set by the government. *Size* is the household size. *Income* denotes the annual total household income, while *OOP* denotes annual total OOP payments by the household.

### Data

We built a panel dataset using administrative data, which was provided by the Chishui Health and Family Planning Bureau and the Chishui Office of Poverty Alleviation and Development.

The Chishui Health and Family Planning Bureau provided NCMS data from its management information system, which covers nearly all (99.96%) rural residents in Chishui from 2014 to 2017. Detailed information is collected on beneficiaries’ demographic characteristics and health care utilization. Demographic characteristics include beneficiaries’ gender, age, household size, and residential area. Health care utilization information includes the name and type of health care facility, the primary diagnosis (based on International Classification of Disease, Tenth Revision codes), and the health care expenses (total medical expenditure, reimbursements, OOP payments) for both outpatient and inpatient treatment. NCMS data is widely used to evaluate the health policy in the previous studies [31–33].

The Chishui Office of Poverty Alleviation and Development provided a Poverty Registry System dataset that contained records of all members of the rural population who had lived or continue to live below the poverty line since 2014. The dataset consists of basic information on each household, including living area, household size, household income per capita, demographic characteristics of household members (such as age and gender), and the cause of poverty. The annual income for the poor households in the Poverty Register System, including income from farming and working as well as informal income, consists of residents’ self-reported income. The declared figure is repeatedly checked by the household members themselves and local government employees. The final income amount of every poor household is made public to every resident living in the same village.

We combined the NCMS management information system data with the Poverty Registry System data using households’ NCMS ID numbers to create a four-year balanced panel dataset from 2014 to 2017. The final four-year panel sample consists of 63,426 households and 253,704 observations (63,426 observations per year * 4 years).

### Empirical strategy

#### Difference-in-difference with propensity score matching (DID-PSM)

We estimated the impacts of the health poverty alleviation project on households’ financial burden (outcome) by combining the DID-PSM methods [14, 34, 35].

All (99.96%) rural households in Chishui during 2014 to 2017 are included in the analysis. We defined poor households identified by the Poverty Registry System as the treatment group and all others as the control group. Thus, we estimate the effect of the project by comparing the pre-post difference in treatment outcome between households that had benefits under health poverty alleviation project and those from the control group.

DID estimation assume that, in the absence of treatment, the change in outcomes between the pre- and post-intervention periods for the treated would be similar to that for the untreated [36]. We compare average changes in outcomes before and after the introduction of the health poverty alleviation project using PSM to control for the initial heterogeneity. Assessing DID without using PSM to adjust for initial conditions between treatment and control groups would result in biases if the initial conditions were influencing placement of the project [34, 37]. As the treatment group for the health poverty alleviation project includes households identified as being poor, they would differ from the control group in their initial conditions. The use of PSM allows the treatment and control groups to be balanced in terms of both their initial conditions and, consequently, their likely improvements in outcomes in the absence of the health poverty alleviation project.

While only individuals experience health shocks, coping with the resultant health financial shock occurs at the household level. Thus, our analyses were conducted at the household level. We also used the individual as the analysis unit, with similar results. Due to space limitations, these results are not reported but are available upon request.

We used the 2014 sample, the starting point of the panel, to estimate the propensity score. The propensity scores were generated using a logit model with the following explanatory variables: (1) Residential areas, a set of dummy variables, including 100 villages in Chishui. (2) Household characteristics, including household size, number of people aged 65 and above, number of people aged 14 and below, and number of males; and (3) Number of diseases in the household in each International Classification of Disease, Tenth Revision, Clinical Modification (ICD-10-CM) chapter, which refers to a set of variables for the 21 total chapters in the ICD-10-CM.

One-to-one nearest-neighbor matching without replacement is used for our main analysis. All units are based on the common support of propensity scores. One-to-four nearest-neighbor matching, radius matching, and kernel matching strategies are also employed in our sensitivity analysis.

After creating comparable treatment and control groups in 2014, we matched households in the years 2015, 2016, and 2017 for comparable and balanced panel data. In the second stage, we estimate the DID models. The model is defined as follows:

2 $$ {y}_{it}=\alpha +\beta {Post}_t+\gamma {Treatment}_i+\lambda \left({Post}_t\bullet {Treatment}_i\right)+{\mathbf{X}}_{it}^{\prime}\boldsymbol{\delta} +{\varepsilon}_{it} $$

where *i* denotes a household, *t* denotes year. *y* denotes the outcome variables, including OOP payments, the occurrence of catastrophic health expenditure, and the occurrence of impoverishing health spending. *Post* is a dummy variable, equal to 1 for a household in 2016–2017 (after the introduction of the project) and 0 otherwise. *Treatment* is a dummy variable, equal to 1 if a household is registered as a poor household and 0 otherwise. **X** is a vector of control variables, including residential areas, household characteristics, number of diseases in the household in each ICD-10-CM chapter, and annual inpatient and outpatient visits in each types of institutions. *ε* denotes the error term.

The coefficient *β* measures the change in the outcome variable between the periods of pre- and postintervention. *γ* captures any difference in outcome variables between the control and treatment groups. The coefficient of interest *λ* for the interaction term *Post×Treatment* measures the change in the outcome variable for the treatment group compared with those of the control group as a result of the project implementation. *δ* measures the change of outcome variable in covariates in household *i* and year *t*. *α* is a constant term.

#### Outcome indicators

In line with the existing literature [38–40], three indicators of the financial burden of illness are used:

1. Total OOP expenditures of the household in the past year (direct medical costs) for outpatient and inpatient services. Considering the positively skewed distribution of this expenditure, we perform a logarithmic transformation for it in the regressions.
2. The occurrence of catastrophic health expenditure. According to the literature [40–43], catastrophic health expenditure in our main analysis is defined as annual OOP payments exceeding 10% of annual household income. The variable is defined as “1” when a household incurs catastrophic health expenditure and “0” otherwise. For the sensitivity analyses, we use an alternative measurement of the catastrophic health expenditure, defined as annual OOP payments exceeding 40% of annual household income. The Poverty Registry System records household income for each household in poverty, but not for the non-poverty households. Thus, we use the average rural household income in Chishui as a proxy for the non-poverty household income when identifying whether a family experienced catastrophic health expenditure.
3. The occurrence of impoverishing health spending. This measure concerns whether OOP payments are forcing a household into poverty or deeper poverty [40, 44, 45]. The occurrence of impoverishing health spending is defined for two cases. The first is the case of a non-poverty household when their disposable income falls below the poverty line after OOP payments. For the poor household, in accordance with the literature [46, 47], we define the occurrence of impoverishing health spending as when annual OOP payments exceed 6% of the annual total household income, which means that illness forces the household into deeper poverty. An impoverishing health spending occurrence is defined specifically as follows:

3 $$ \mathrm{Impoverishing}\ \mathrm{health}\ \mathrm{spending}=\left\{\begin{array}{c}1=\left\{\begin{array}{c}\mathrm{OOP}>\mathrm{Income}- PL\times Size,\mathrm{if}\ \mathrm{Income}> PL\times Size\\ {} OOP>6\%\mathrm{Income},\kern0.5em \mathrm{if}\kern0.5em \mathrm{Income}\le PL\times Size\end{array}\right.\\ {}0= Otherwise\end{array}\right. $$

where *OOP* denotes the annual total OOP payments of the household. *Income* denotes the annual total household income. *Size* is the household size. *PL* is the national poverty line. The national poverty line in China was 2800 RMB per year (approximately $1 per day) in 2014 and 2015 and 3146 RMB per year (approximately $1.26 per day) in 2016 and 2017.

#### Control variables

As recommended in the literature [12, 35, 48], we include a series of control variables in the DID regression analysis to adjust for potential confounders in the relationship between the implementation of the health poverty alleviation project and the financial burden of illness. The control variables are the following: (1) Residential areas, a set of dummy variables, including 100 villages in Chishui. There would be a potential problem in estimation if a large number of dummy variables were to be included in the model. We have used the township dummies to replace the villages in the regressions. This reduce the number of dummy variables reduced from 99 to 16. The effects are still statistically significant after the replacement, but attenuate somewhat. The reduction in effects could be caused by neglecting the differences between poor and non-poor villages. As a result, we controlled the village dummies. (2) Household characteristics, including household size,^1^ number of people aged 65 and above, number of people aged 14 and below, and number of males per household. (3) Number of diseases from each ICD-10-CM chapter in the household. (4) Annual inpatient visits in each type of health institution (township health center, hospital in Chishui, and hospital outside Chishui) per household. (5) Annual outpatient visits in each type of institution (village clinic, township health center, and hospital) per household.

#### Decomposing the effects of the health poverty alleviation project

The Chishui government extended the social security system from three into five levels, to supplement the benefit packages of the health alleviation project. As a result, we examine whether the extra packages have enhanced the effect on financial risk protection for poverty households.

This assessment is conducted by decomposing the whole project into three components: (1) The national basic service packages, including the prevention program, treatment capacity building, and the first three levels of the social security system; (2) specialized commercial health insurance, or the fourth level of the social security system; and (3) the specific subsidy, which is the fifth level of the social security system.

The DID model, as in eq. (2), is employed in a decomposing process. We begin by analyzing the effect of component (1) based on OOP whereby reimbursement from component (1) is the outcome variable. Second, we estimate the effect of both component (1) and (2) based on OOP whereby reimbursements from component (1) and (2) are the outcome variable. Then, the effect of component (2) is calculated as the effect of the component (1) and (2) minus the effect of component (1). Third, we estimate the effect of the whole package, using OOP based on reimbursement from the whole package as the outcome variable. The effect of component (3) is calculated as the effect of the whole package minus component (1) and (2). The decomposing process of the other two outcome variables (The occurrence of catastrophic health expenditure and impoverishing health spending) are same as decomposing OOP.

The analysis in our study is carried out using STATA version 14.1.

#### Ethics

This study has been approved by the ethics committee of Sichuan University, China, and the approval number is K2018087. The data used in our analysis are administrative data. All the personal privacy information has been desensitized before analysis.

## Results

### PSM results and sample

**Fig. 6:**
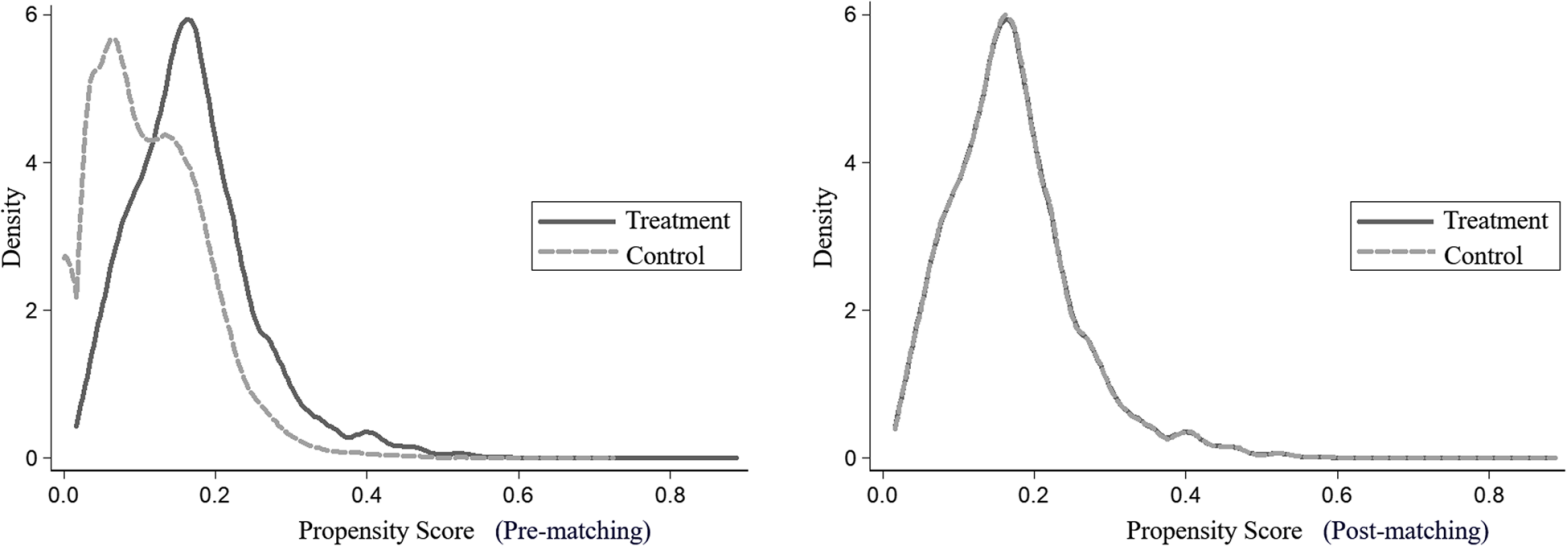
PSM in full sample in 2014. Notes: the graph on the left is for samples before matching, and that on the right is for samples after matching. The solid lines plot scores for the treatment group, while the dashed lines plot scores for the control group

Figure 6 depicts the quality of matching in our sample. After matching, samples featured all regions of overlap and readily passed the balancing tests, showing effective matching of poor and nonpoor households with similar characteristics on the observed variables.

There are 7610 households each in the treatment and control groups. Each household is observed for 4 years; thus, we have a total of 60,880 observations in our final panel samples during the four-year study period. The payment records in the NCMS system are complete; there is no missing data regarding co-payments and total payments.

For the control variables, data are missing for 1.42% of the residential area. To control for this in the regressions, we added a variable indicating whether the residential area data were missing. Data are missing for 5.42% of patient’s diagnose. Hence, we also added a variable to indicate missing diagnosis data in our regression models to control for this issue.

### Descriptive statistics

Table 1 presents descriptive statistics for key variables for the treatment and control groups pre- and post-intervention. OOP payments for both groups decrease in the post-intervention period. Regardless of the period, OOP payments in poor households are lower than in non-poor households. However, poor households incur more catastrophic and impoverishing health expenditures than do non-poor households. With the implementation of the health poverty alleviation project, the proportion of poor households with catastrophic or impoverishing health spending decreased. Regarding household characteristics, poor households have larger household sizes and include more elderly members than do non-poor households, while the number of children per household in both groups is similar. For health care utilization, poor households are more likely to utilize inpatient care in the township health center and hospital in Chishui. Outpatient care was sought more frequently from the township health center, in both pre- and postintervention periods.

**Table 1:** Description of key variables

Variables	Treatment group	Control group	Statistic	*p*
	Mean	Mean		
OOP payments				
Preintervention	369.920	438.992	−4.843^a^	<0.001
Postintervention	250.737	315.442	−6.143^a^	<0.001
Occurrence of catastrophic health expenditure				
Preintervention	0.143	0.070	420.074^b^	<0.001
Postintervention	0.070	0.063	4.660^b^	0.031
Occurrence of impoverishing health spending				
Preintervention	0.138	0.015	1600^b^	<0.001
Postintervention	0.017	0.007	69.412^b^	<0.001
Number of Males per household				
Preintervention	1.742	1.697	−0.024^a^	0.981
Postintervention	1.754	1.713	−1.292^a^	0.196
Household size				
Preintervention	3.568	3.472	−4.476^a^	<0.001
Postintervention	3.589	3.498	−4.210^a^	<0.001
Number of people aged 65 and above per household				
Preintervention	0.867	0.850	−2.371^a^	0.017
Postintervention	0.997	0.963	−3.920^a^	0.001
Number of people aged 14 and below per household				
Preintervention	0.581	0.555	−1.804^a^	0.071
Postintervention	0.469	0.463	0.513^a^	0.608
Annual inpatient visits in each types of institutions per household				
Preintervention				
Township health center	0.121	0.103	−3.243^a^	0.001
Hospital in Chishui	0.312	0.263	−3.690^a^	<0.001
Hospital out of Chishui	0.094	0.086	−0.159^a^	0.873
Postintervention				
Township health center	0.233	0.160	−8.007^a^	<0.001
Hospital in Chishui	0.475	0.342	−10.018^a^	<0.001
Hospital out of Chishui	0.080	0.085	3.569^a^	<0.001
Annual outpatient visits in each types of institutions per household				
Preintervention				
Village clinic	2.553	2.600	−2.960^a^	0.003
Township health center	1.183	0.958	−3.690^a^	<0.001
Hospital	0.139	0.112	− 0.159^a^	0.873
Postintervention				
Village clinic	2.619	2.772	−1.729^a^	0.083
Township health center	1.417	1.140	−10.018^a^	<0.001
Hospital	0.117	0.104	3.569^a^	<0.001

Notes on superscripts: (1) “a” denotes the statistic of Mann–Whitney U test. (2) “b” denotes the statistic of Chi-square test

### Effects of the health poverty alleviation project on financial risk protection

Table 2 presents the regression estimations for the impact of the health poverty alleviation project reflecting the three aspects of financial risk protection: OOP payments, catastrophic health expenditure, and impoverishing health spending. The estimations of the interaction term *Post×Treatment* capture the impact of the health poverty alleviation project. The estimated coefficients of the interaction term for the three regressions (OOP payments, catastrophic health expenditure, and impoverishing health spending) are − 0.150, − 0.077, and − 0.117, respectively. All these values are statistically significant (*p < 0.01*). The results indicate that the health poverty alleviation project led to an average reduction in OOP payments, and lower probability of incurring catastrophic or impoverishing health expenditure by 15.0, 7.7, and 11.7%, respectively. This constitutes a significant improvement in financial risk protection, signaling the success of the health poverty alleviation project.

**Table 2:** Effects of the health poverty alleviation project on financial risk protection

Variables	OOP payments	Occurrence of catastrophic health expenditure	Occurrence of impoverishing health spending
*Post*×*Treatment*	− 0.150***	− 0.077***	− 0.117***
	(0.037)	(0.004)	(0.003)
*Post*	0.004	− 0.019***	−0.007***
	(0.029)	(0.002)	(0.001)
*Treatment*	0.091***	0.066***	0.120***
	(0.027)	(0.003)	(0.000)
Control variables	Yes	Yes	Yes
*R* ^*2*^	0.643	0.367	0.228
Observations	60,880	60,880	60,880

Notes: (1) *** denotes *p* < 0.01, ** denotes *p* < 0.05

(2) Robust standard errors in parentheses

(3) The control variables include residential areas, household size, numbers of elderly members, males and children per household, number of diseases in each ICD-10-CM chapter per household, and annual inpatient and outpatient visits in each type of institutions per household

(4) The estimates of occurrence of catastrophic health expenditure and impoverishing health spending are marginal effects

### Effects across different quantiles of poor households

We also explore the effects of the health poverty alleviation project across different quantiles of households in poverty. We categorize the poor households by income into three quantile groups: The first tercile group, Q1 represents 33.3% of the population with the lowest income among the poor; the second tercile group Q2, represents 33.3% of the population with the middle income among the poor; and the third tercile group Q3, represents 33.3% of the population with the highest income among the poor. Thus, we have four groups, Q1, Q2, Q3, for three quantiles of the poverty households, and non-poverty households.

Table 3 reports the quantile analysis results of poor households. The results imply that households in the first tercile would be the households with the highest financial risk protection. This shows that the health poverty alleviation project effectively targeted the poorest of the poor.

**Table 3:** Effects across different quantiles of poor households

Variables	OOP payments	Occurrence of catastrophic health expenditure	Occurrence of impoverishing health spending
*Treatment*	0.089**	0.096***	0.179***
	(0.038)	(0.004)	(0.005)
*Post*	0.004	−0.011***	−0.007***
	(0.030)	(0.002)	(0.001)
*Treatment* ×*Post*	−0.262***	−0.108***	− 0.176***
	(0.053)	(0.006)	(0.005)
*Q2* × *Treatment×Post*	0.119	0.031***	0.030***
	(0.064)	(0.007)	(0.007)
*Q3* × *Treatment×Post*	0.218***	0.064***	0.147***
	(0.063)	(0.007)	(0.006)
*Q2*	−0.075	−0.023***	−0.030***
	(0.045)	(0.006)	(0.007)
*Q3*	0.082	−0.065***	−0.148***
	(0.045)	(0.005)	(0.006)
Control variables	Yes	Yes	Yes
*R* ^*2*^	0.644	0.380	0.252
Observations	60,880	60,880	60,880

Notes: (1)*** denotes *p* < 0.01, ** denotes *p* < 0.05

(2) Robust standard errors in parentheses

(3) The control variables include residential areas, household size, numbers of elderly members, males and children per household, number of diseases in each ICD-10-CM chapter per household, and annual inpatient and outpatient visits in each type of institutions per household

(4) The estimates of occurrence of catastrophic health expenditure and impoverishing health spending are marginal effects

### Effects on total medical expenses and health care utilization

Previous studies have suggested that poor individuals who cannot afford health care thus report lower or no OOP payments [14, 45, 49]. To better understand the empirical results, in this section, we explore the impact of the health poverty alleviation project on health care utilization among households in poverty.

Following the literature [50, 51], we measure health care utilization using the number of inpatient and outpatient health care visits by a household in a year, as well as the total medical expenses. The latter includes OOP payments and reimbursements by health insurance and other financing programs for all inpatient and outpatient services used by a household in a year. We estimate the DID with PSM regressions using the same methods as those described earlier for the financial burden of illness outcome variables. Table 4 presents the estimation results. As shown in Table 4, we find that the health poverty alleviation project increases the annual number of hospitalizations per household by 0.035.

**Table 4:** Effects of health poverty alleviation project on total medical expenses and health care utilization

Variables	Total medical expenses	Number of inpatients	Number of outpatients
*Post*×*Treatment*	0.064	0.035***	−0.012
	(0.046)	(0.011)	(0.048)
*Post*	0.034	−0.010	0.081**
	(0.036)	(0.009)	(0.034)
*Treatment*	0.132***	0.033***	−0.063**
	(0.034)	(0.008)	(0.031)
Control variables	Yes	Yes	Yes
*R* ^*2*^	0.578	0.696	0.746
Observations	60,880	60,880	60,880

Notes: (1) *** denotes *p* < 0.01, ** denotes *p* < 0.05

(2) Robust standard errors in parentheses

(3) The control variables of total medical expenses include residential areas, household size, numbers of elderly members, males and children per household, number of diseases in each ICD-10-CM chapter per household, and annual inpatient and outpatient visits in each types of institutions per household

(4) The control variables of health care utilization include residential areas, household size, numbers of elderly members, males and children per household, number of diseases in each ICD-10-CM chapter per household, and number of household members in each inpatient or outpatient institution

### Decomposing the effects of the health poverty alleviation project

Table 5 presents the decomposition results. The results show that the national basic service packages account for 86.0, 99.1, and 100% of the entire impact on the reduction in OOP payments, and the probability of incurring catastrophic or impoverishing health expenditure, respectively. Levels 4 and 5 of the social security system mainly impact the OOP payments made by households in poverty, accounting for 9.3 and 4.7% of the decline in OOP payments, respectively. However, these payments have little effect on the probability of catastrophic health expenditure and the probability of impoverishing health spending.

**Table 5:** Decomposing the effects of the health poverty alleviation project

Intervention	OOP payments		Occurrence of catastrophic health expenditure		Occurrence of impoverishing health spending	
	DID estimator	Percentage	DID estimator	Percentage	DID estimator	Percentage
Total	−0.150	100%	−0.077	100%	−0.117	100%
National basic service packages	−0.129	86.0%	−0.076	99.1%	−0.117	100%
Specialized commercial health insurance	−0.014	9.3%	−0.001	0.5%	−0.000	0%
Specific subsidy	−0.007	4.7%	−0.000	0.4%	−0.000	0%

## Discussion

The present study explores the financial risk protection effect of the health poverty alleviation project in China. Our results show that the health poverty alleviation project reduces OOP payments and the probabilities of incurring catastrophic or impoverishing expenditure. The results suggest that the health poverty alleviation project is achieving its goal of providing financial risk protection against illness for poor populations in China.

### Reasons for effectiveness of financial risk protection

First, targeting households in poverty under the health poverty alleviation project is one of the main reasons for its impact on financial risk protection. Although the basic health insurance program— NCMS—achieved nearly universal coverage of rural residents in China in 2010, the benefit package is not generous because premiums is set at a low level. After regular reimbursement from NCMS, OOP payments still represent a great financial burden to rural households with relatively low incomes, especially the poor [16, 52–55]. To address this problem, the health poverty alleviation project enhances the NCMS benefit package for households in poverty and provides them with special medical aid. Through these targeted programs, the health poverty alleviation project has effectively decreased the financial burden of ill health for the poor.

Second, the high degree of accountability of the management system and interdepartmental coordination among government departments are also factors in the effectiveness of the health poverty alleviation project. In China, social health insurance programs and medical aid are accountable to different government departments. In general, NCMS is operated by the Department of Health and Family Planning, while medical aid is administered by the Department of Civil Affairs. Without tight coordination mechanisms that align various priorities among different departments, the overall program would not achieve the goal of improving financial risk protection as effectively [56]. However, the series of management systems developed by the Targeted Poverty Alleviation have ensured interdepartmental coordination to guarantee further efficiency improvements in financial risk protection for households in poverty.

### Comparison with related studies

Studies on the effects of poverty alleviation programs on OOP payments and health care utilization have mainly presented two results: One is that OOP payments decrease with less health care utilization after the program [14, 45, 49], and the others report an offsetting effect, showing increasing health care utilization but either a small or no effect on OOP payments [12, 35, 57, 58]. Our findings show that China’s health poverty alleviation project not only reduces OOP payments for poor households but also increases inpatient health care utilization, indicating an intensive effect on financial risk protection.

Lacking financial accessibility, the poor tend to suffer from having a lower level of health care utilization, despite their higher levels of needs [59]. According to China’s National Health Service Survey, in 2013, 20.7% of rural poor patients who should have been hospitalized were not due to lack of affordability [60]. Due to the improved financial capability granted by the project, household in poverty were able to respond to demand for hospital care.

### Sensitivity analysis

Applying different matching strategies may affect the results. To test the sensitivity of our results to different choices of the PSM matching strategy, 1:4 nearest-neighbor matching, radius matching, and kernel matching are employed. The results (see Table 6) are similar using the three alternative matching strategies, thus demonstrating the robustness of our main results.

**Table 6:** Effects of health poverty alleviation calculated by different matching strategies

Variables	OOP payments			Occurrence of catastrophic health expenditure			Occurrence of impoverishing health spending		
	1:4 nearest	Radius	Kernel	1:4 nearest	Radius	Kernel	1:4 nearest	Radius	Kernel
*Post*×*Treatment*	−0.229***	−0.099***	− 0.096***	−0.076***	− 0.073***	−0.072***	− 0.116***	−0.115***	− 0.114***
	(0.030)	(0.028)	(0.028)	(0.003)	(0.003)	(0.003)	(0.003)	(0.003)	(0.003)
*Post*	0.022	−0.084***	−0.082***	−0.017***	− 0.019***	−0.019***	− 0.008***	−0.008***	− 0.008***
	(0.015)	(0.011)	(0.011)	(0.001)	(0.001)	(0.001)	(0.001)	(0.000)	(0.000)
*Treatment*	0.128***	0.031	0.034	0.066***	0.063***	0.064***	0.120***	0.121***	0.121***
	(0.021)	(0.020)	(0.020)	(0.002)	(0.002)	(0.002)	(0.003)	(0.003)	(0.003)
Control variables	Yes	Yes	Yes	Yes	Yes	Yes	Yes	Yes	Yes
*R* ^*2*^	0.663	0.655	0.653	0.379	0.370	0.369	0.207	0.189	0.188
Observations	136,544	239,904	239,912	136,544	239,904	239,912	136,544	239,904	239,912

Notes: (1) *** denotes *p* < 0.01, ** denotes *p* < 0.05

(2) Robust standard errors in parentheses

(3) The control variables include residential areas, household size, numbers of elderly members, males and children per household, number of diseases in each ICD-10-CM chapter per household, and annual inpatient and outpatient visits in each types of institutions per household

(4) The estimates of occurrence of catastrophic health expenditure and impoverishing health spending are marginal effects

Considering that there are different thresholds for defining catastrophic health expenditure, the estimated effect differs among the various thresholds. We also use OOP payments exceeding 40% of annual household income as an alternative measurement to test the sensitively of our results. The estimations (see Table 7) are similar, confirming the robustness of the definition.

**Table 7:** Effects on occurrence of catastrophic health expenditure at 40% thresholds by different matching strategies

Variables	Occurrence of catastrophic health expenditure			
	1:1 nearest without replacement	1:4 nearest	Radius	Kernel
*Post*×*Treatment*	−0.034***	−0.037***	−0.037***	−0.033***
	(0.002)	(0.002)	(0.002)	(0.002)
*Post*	−0.012***	−0.009***	− 0.012***	−0.012***
	(0.001)	(0.001)	(0.001)	(0.001)
*Treatment*	0.033***	0.035***	0.033***	0.033***
	(0.002)	(0.002)	(0.002)	(0.002)
Control variables	Yes	Yes	Yes	Yes
*R* ^*2*^	0.210	0.200	0.195	0.194
Observations	60,880	136,544	239,904	239,912

Notes: (1) *** denotes *p* < 0.01, ** denotes *p* < 0.05

(2) Robust standard errors in parentheses

(3) The control variables include residential areas, household size, numbers of elderly members, males and children per household, number of diseases in each ICD-10-CM chapter per household, and annual inpatient and outpatient visits in each types of institutions per household

### Policy implication

Our study highlights that health poverty alleviation provides financial risk protection for the poor. This means the policy should be continued.

Meanwhile, the decomposing results imply that most of the overall effect leading to the improvement of financial risk protection is attributable to the national basic service packages, while the extended packages from the Chishui government have a complementary effect by releasing the economic burden faced by households in poverty. In other words, our study shows that the national basic service packages have sufficient impact to achieve the goal of strengthening financial risk protection against illness among poor populations. This indicating that the local government should carefully consider any enrichments to the benefit package they offer.

This study also provides important implications for the ongoing discussions on reforming the Chinese health financing system and contributes to the international literature on alleviating poverty through strengthening financial risk protection against illness.

### Limitations

The main limitation of this study is regarding its generalizability. As only data from Chishui City are used for this empirical analysis, generalizations of our estimations should be made with care. The provision of the unified benefit package of the health poverty alleviation project is suggested by the Central Government. However, local governments are authorized to enrich the benefit package depending on their own situation. Chishui extended the three-level national social security system into five-levels. The five-level protection against the economic burden of illness is therefore enhanced. As the implementation capacity of local governments differs among regions, there would be an impact on the policy effects.

Second, we do not include the direct non-medical costs associated with care-seeking (e.g., informal payment) when estimating OOP payments. If informal payments were not changed along with the policy implementation, the pre- and post-informal payments would offset each other in the DID (differences in difference) setting. In this case, our estimation of policy impact would be consistent. However, if informal payments either rose or fell, our estimation would under- or overestimate the policy impact, respectively. Further researches should consider include informal payments if data available.

Third, due to data limitations, we do not further explore the effect of health poverty alleviation on the sub item of OOP, such as OOP for drug, OOP for diagnosis. This will be an important topic for future research.

Fourth, our dataset from NCMS did not include income data of non-poor households. We used the average rural household income in Chishui as a proxy for the non-poor household income when identifying whether a family experienced catastrophic health expenditure. Future research should consider the exact income data of all residents. Survey data could also be used to further explore the effect.

Fifth, due to the four-year study period, we are only able to evaluate the short-term (2 year) impact of the health poverty alleviation project. Future studies using provincial or national datasets with more detailed information over a longer period may improve our estimations.

## Conclusion

The health poverty alleviation project, an integral component of the Targeted Poverty Alleviation in China, was implemented to strengthen financial risk protection against illness for poor populations. Our study demonstrates that the health poverty alleviation project significantly improves financial risk protection by reducing OOP payments and decreasing the probabilities of catastrophic or impoverishing health expenditure. Our study provides important implications for ongoing discussions on reforming the Chinese health financing system and poverty reduction policies.

## Data Availability

The data that support the findings of this study are available for the Chishui Health and Family Planning Bureau and the Chishui Office of Poverty Alleviation and Development but restrictions apply to the availability of these data, which were used under license for the current study, and so are not publicly available. Data are however available for the authors upon reasonable request and with permission of the Chishui Health and Family Planning Bureau and the Chishui Office of Poverty Alleviation and Development.

## Abbreviations

CPC: Communist party of China
DID: Difference-in-differences
HER: Electronic health records
ICD-10-CM: International classification of disease, tenth revision, clinical modification
NCMS: New Rural Cooperative Medical Scheme
NGOs: Nongovernmental organizations
OOP: Out-of-pocket
PSM: Propensity score matching

## Footnotes

1 The household size would be a confounder in the analysis of the policy impact on medical expenses. Controlling it in the regression model or dividing the outcome indicators by household size are two means of adjusting for the confounder. The results estimated by the two approaches are similar. Considering that two of our main indicators (the occurrence of catastrophic health expenditure, and the occurrence of impoverishing health spending) could not be divided by household size, our main manuscript represents the results by controlling household size in the regressions.
